# Retraction: Differential Expression of the RANKL/RANK/OPG System Is Associated with Bone Metastasis in Human Non-Small Cell Lung Cancer

**DOI:** 10.1371/journal.pone.0314046

**Published:** 2024-11-13

**Authors:** The PLOS ONE Editors

After this article [1] was published, concerns were raised about Figs 1, 2, and 6.

Specifically:

- In Fig 1A, the lower right region of the PG-LH7 panel appears similar to the upper left region of the PAa panel.
- The MMP9 panel in Fig 1C appears similar to the OPG panel in Fig 2A, when rotated 180 degrees and with altered aspect ratio.
- The “GADPH” panel in Fig 1C and the middle and lower “GADPH” panels in Fig 2A appear similar.
- The lower left region of the middle RANKL–Bone Metastases panel in Fig 6A appears similar to the upper right region of the middle OPG–Bone Metastases panel in Fig 6A.

During editorial follow-up, the corresponding author WG stated the MMP9 panel in Fig 1C and the OPG panel in Fig 2A originated from separate PCR experiments. The corresponding author also stated that the underlying data for Figs 1C and 2A are no longer available. They did not respond or could not be reached for comment on the additional concerns.

The above issues raise concerns about the reliability of the reported results and conclusions; in the absence of the original data these issues cannot be resolved and the *PLOS ONE* Editors retract this article.

WG did not respond directly to the editorial decision. XP, TR, ZL, XL, SZ, QL, and YS either did not respond directly or could not be reached.
